# Food & Nutrition Research with new aims and scope

**DOI:** 10.29219/fnr.v62.1396

**Published:** 2018-04-26

The *Food & Nutrition Research* (FNR) journal is owned by the Swedish Nutrition Foundation (SNF), a non-profit organisation. SNF was founded in 1961 with the aim of promoting nutrition-related research and its practical application. To this end, the FNR leadership made the bold decision in 2008 to begin publishing electronically as an open access journal and to cease with the printed version. This has accelerated worldwide online distribution and access to the FNR for both authors and readers.

Although the Open Access movement has exponentially increased the number of high-quality journals, there are simultaneous increases in low-quality journals. However, the strong rise in popularity of citation frequency, impact factors and valued credibility component provided by reputable journals prevent the surge of this sort of open-access journals.

FNR is now well established as a peer-reviewed, open access journal that strives to briskly review, publish and disseminate high-quality research in nutritional sciences. FNR has recently decided to change the overall focus of the journal to different aspects of human nutrition. The new aims and scope include nutrition ranges from whole-body metabolism, clinical health, fitness and population health and deep mechanistic studies at molecular/biochemical and cellular levels relevant to human nutrition. This scope directly supports SNF’s mission to extend the knowledge of nutrition through basic, multidisciplinary and clinical research to improve public health and clinical practice. Areas of new and added emphasis include the following: eating behaviour and qualitative assessments; nutrition education; implementation; nutrition and exercise physiology; nutritional impacts on cognitive function; intervention programme methods and outcomes; evaluation and validation studies of dietary and nutrition methods; food and nutrition policy; and food processing, packaging and storage affecting nutritional aspects.

The major changes for the journal are simplified below:

- A revised scope encompassing the breadth of human nutrition research
- A simplified submission process with the new publishing partner Open Academia

The changes in scope demand an expansion of editorial structure to provide the breadth of expertise necessary to chaperone high-quality and rapid peer review. Accordingly, the FNR is now restructured with additional editors with broader expertise in human nutrition and a statistical advisor.

I am really excited with the new expanded editorial team and the new publishing partner, Open Academia.

I look forward to working with the nutrition community to ensure continued success of this journal.

*Professor Asim K. Duttaroy* Editor-in-Chief

